# A new molecular breast cancer subclass defined from a large scale real-time quantitative RT-PCR study

**DOI:** 10.1186/1471-2407-7-39

**Published:** 2007-03-05

**Authors:** Maïa Chanrion, Hélène Fontaine, Carmen Rodriguez, Vincent Negre, Frédéric Bibeau, Charles Theillet, Alain Hénaut, Jean-Marie Darbon

**Affiliations:** 1 INSERM U868, Cancer Research Centre, CRLC Val d'Aurelle-Paul Lamarque, Montpellier, France; 2 UMS 2293 CNRS, University of Evry-Val d'Essonne, France

## Abstract

**Background:**

Current histo-pathological prognostic factors are not very helpful in predicting the clinical outcome of breast cancer due to the disease's heterogeneity. Molecular profiling using a large panel of genes could help to classify breast tumours and to define signatures which are predictive of their clinical behaviour.

**Methods:**

To this aim, quantitative RT-PCR amplification was used to study the RNA expression levels of 47 genes in 199 primary breast tumours and 6 normal breast tissues. Genes were selected on the basis of their potential implication in hormonal sensitivity of breast tumours. Normalized RT-PCR data were analysed in an unsupervised manner by pairwise hierarchical clustering, and the statistical relevance of the defined subclasses was assessed by Chi2 analysis. The robustness of the selected subgroups was evaluated by classifying an external and independent set of tumours using these Chi2-defined molecular signatures.

**Results:**

Hierarchical clustering of gene expression data allowed us to define a series of tumour subgroups that were either reminiscent of previously reported classifications, or represented putative new subtypes. The Chi2 analysis of these subgroups allowed us to define specific molecular signatures for some of them whose reliability was further demonstrated by using the validation data set. A new breast cancer subclass, called subgroup 7, that we defined in that way, was particularly interesting as it gathered tumours with specific bioclinical features including a low rate of recurrence during a 5 year follow-up.

**Conclusion:**

The analysis of the expression of 47 genes in 199 primary breast tumours allowed classifying them into a series of molecular subgroups. The subgroup 7, which has been highlighted by our study, was remarkable as it gathered tumours with specific bioclinical features including a low rate of recurrence. Although this finding should be confirmed by using a larger tumour cohort, it suggests that gene expression profiling using a minimal set of genes may allow the discovery of new subclasses of breast cancer that are characterized by specific molecular signatures and exhibit specific bioclinical features.

## Background

Breast cancer is the most common female cancer in the Western world and the leading cause of death by cancer among women [1]. It is a complex genetic disease characterized by an accumulation of molecular alterations resulting in an important clinical heterogeneity. Current prognostic factors (including lymph node status, tumour size, histological grade, hormone receptor status, ERBB2 expression and patient age) are insufficient to accurately predict the clinical outcome. High-throughput molecular technologies, including large-scale RT-PCR and cDNA microarrays, have made possible to study the gene expression profiles of tumours. Unsupervised analysis of data by hierarchical clustering allows grouping tumours on the basis of similarities in their gene expression patterns. Samples that share molecular profiles might be expected to share phenotypic features, such as those that can define the severity of the disease. Hierarchical clustering of gene expression patterns has been successfully used to identify subtypes of breast tumours that exhibit distinct clinical behaviours [2-6]. At least five subtypes (luminal A, luminal B, basal-like, ERBB2, and normal-like) have been identified on the basis of the pattern of expression of a 500-gene set. The luminal A and luminal B subtypes gather ER+ tumours, while the basal-like, ERBB2 and normal-like subclasses assemble ER- tumours. Interestingly, the luminal subtype A exhibits a relatively good prognosis, while the luminal B tumours present a worse prognosis. The basal-like and ERBB2 subsets show the worst clinical outcome [3,4]. This molecular classification has been confirmed using extended or different tumour sets [4], as well as partly distinct or reduced gene sets [4-6].

Noteworthy, a similar taxonomy of breast cancers has been characterized using immunohistochemistry [7-9], although further work seems necessary to correlate the respective subtypes at mRNA and protein expression levels.

However, more than 30% of the 295 breast tumours, which have been used to identify and validate the 70-gene good prognosis signature [10,11], could not be confidently assigned to any of the five subtypes defined so far [12]. Such an inability to classify all breast cancers in the five molecular subtypes may be due to an incomplete representation of the genes used for the intrinsic set of genes (when compared to the initial one) or, alternatively, to the distinct nature of the tumours used in the different studies. In any case, this failure suggests that other molecular subclasses are waiting for characterization.

In the present study, we have classified 199 primary breast tumours and 6 normal breast tissues based on the expression of 47 genes that had been selected on the basis of their possible involvement in breast tumour hormonal sensitivity. Gene expression was evaluated by measuring levels of specific mRNAs using quantitative RT-PCR. Following hierarchical clustering and Chi2 analysis of the expression data, we defined a series of molecular breast cancer subgroups that were characterized by specific molecular signatures. They are either reminiscent of those previously reported, or represent putative new subclasses. One of the subtypes, which we defined, gathered tumours with specific bioclinical features including a low rate of recurrence within a 5 year follow-up.

## Methods

### Patients and breast tissue samples

A total of 199 primary breast carcinomas and 6 normal breast tissues were analysed in this study. They were obtained from patients who had undergone initial surgery at the Cancer Research Centre Val d'Aurelle-Paul Lamarque in Montpellier. All tumours were from patients who did not receive neo-adjuvant treatment. The patients' age at diagnosis varied from 27 to 92 years (mean 63 years, median 65 years). All but 1 patient were treated with one or more adjuvant therapies (Additional File 1, Table S1). This study was conducted under the approval of the Institutional Review Board of the Cancer Research Centre Val d'Aurelle-Paul Lamarque. Informed consent was obtained from the patients prior to surgery. For the 199 patients, the median follow-up time was 65.4 months. Recurrence was observed in 34 patients (27 distant and 5 local recurrences, 2 not determined). The median recurrence time was 32.3 months.

Fresh tissues were processed immediately after surgical removal. One part of each tumour was formalin-fixed and paraffin-embedded to establish the histological type (139 ductal and 35 lobular carcinomas, 10 mixed ductal/lobular carcinomas and 15 other types; Additional File 1, Table S2) and the histological grade (WHO classification: 16% SBR I, 55% SBR II and 26% SBR III tumours; Additional File 1, Table S3). Lymph nodes were also available (38% patients were N+ at the time of diagnosis, Additional File 1, Table S3). The remaining of each tumour was snap-frozen in liquid nitrogen and stored at -80C. Frozen sections were stained with Haematoxylin and Eosin and analysed by an experienced breast pathologist. Eligible samples had to consist of at least 50% of tumour cells. ER status was determined by using ligand-binding assay (the ER positivity threshold was ≥ 10 fmol/mg).

### RNA extraction and purification

Frozen breast samples were homogenized using the FastPrep System from Q-Biogene. Briefly, approximately 40 mg of frozen tissues were broken up in lysing buffer on a lysing matrix for 40 sec. Total RNA was extracted and cleaned up from the lysate using the Qiagen Rneasy Mini Kit. The RNA purity and integrity was controlled by way of the Bioanalyser 2100 from Agilent. Only RNAs with a score 8–10 were included in this study.

### cDNA synthesis

After DNAse treatment, 1 μg of total RNA was incubated with 250 ng of random hexamer for 10 min at 70°C. Total RNA was reverse transcribed in a final volume of 20 μl containing 1× first strand buffer, 0.1 M DTT, 10 mM dNTP and 200 units of Superscript RT. The samples were incubated at 25°C for 10 min, and then at 42°C for 1 h. The reverse transcriptase was finally inactivated by heating at 70°C for 15 min.

### PCR amplification

Primers of the selected genes were designed using the Primer Express software (PE Applied Biosystems), based on published sequences, and oligonucleotides were obtained from Proligo.

For quantitative RT-PCR, 2 μl of diluted RT-reaction samples (1/15) were added to 13 μl of a PCR mixture made up of 7.5 μl of 2× SYBR Green PCR Master Mix (Applied Biosystems), 0.075 μl of each primer at a concentration of 100 μM and RNAse-free water. The thermal cycling conditions comprised an initial step at 50°C for 2 min and a denaturation step at 95°C for 10 min, followed by 40 cycles at 95°C for 15 sec and 60°C for 1 min. All PCR reactions were carried out using an ABI Prism 7000 Sequence Detection System (Applied Biosystem). The specificity of each primer couple was demonstrated by a dissociation curve analysis. To generate a calibration curve, a serially diluted cDNA mixture was used as standard and quantified for each primer set. The standard concentration was plotted against the cycle number at which the fluorescence signal increased above the background (threshold) value (Ct value). The amplification efficiency, E (%) = (10^(1/-s)^-1)*100 (s = slope), of each standard curve was determined and appeared to be > 95% and < 105%, over a wide dynamic range.

### Unsupervised hierarchical clustering of the Q-RT-PCR data

The 205 breast samples were distributed in three separate 96-well blocks, according to the time of sample processing. For each experimental sample, the amount of the gene of interest and of 28S, the endogenous reference, was determined from the appropriate standard curve in independent experiments. Measurements were performed in duplicate for each data point and those with a coefficient of variation for the Ct value > 0.5 were tested again. We calculated the relative fold-change using the comparative cycle times (Ct) method with 28S as a reference. The expression value of each gene in each tumour sample was normalised to the mean expression value for that gene in all the samples in the block in such a way that each block had the same overall expression value for one given gene.

Unsupervised analysis of the data was applied to investigate the relationships among genes and among samples. Hierarchical pairwise average-linkage clustering was performed by means of the Cluster and TreeView software [13], using Log2-transformed data, median-centered gene expression values and Pearson correlation as similarity metrics.

### Chi2 statistical analysis

The classification parameter, which was chosen to assess the statistical relevance of the subgroups defined by hierarchical clustering, was based on the threshold values of gene expression. Theoretically, for each relevant gene, all the samples from one subgroup and those from the others should be, respectively, below or above a defined threshold. The optimal threshold, which allowed the best discrimination, was defined by a Chi2 analysis.

Firstly, we transformed continuous variables (i.e. gene expression intensities) into discrete variables (i.e. number of tumours belonging to a gene expression class, for each gene and for each tumour subgroup). Gene expression classes were set from -4 to +5 by step of 0.1. Then, the Chi2 values were calculated for each of these classes and for each tumour subgroup as indicated in Table 1.

The highest Chi2 among the different classes for each tumour subgroup was used to define the thresholds in order to best discriminate a tumour subgroup from another. The gene-threshold couple was considered able to discriminate one class from the others with a good statistical accuracy, when the corresponding Chi2 value was ≥ 15 (p value ≤ 10^-4^). Thus, to optimize the test and to cut the noise, only Chi2 values ≥ 15 as well as the lowest and highest thresholds among the different subgroups were considered (Additional File 1, Table S4).

By doing so, a molecular signature was assigned to each tumour subgroup. A molecular signature was composed by the genes selected by the Chi2 test with each gene associated to an expression threshold. In that way, each subgroup was characterized by the expression levels of the signature-genes that specify that subgroup. A tumour was classified into the subgroup where its gene expression profile followed the thresholds defined in the signature. For each gene, which specifies one given subgroup, a score of 1 (vs 0) was attributed when the expression level of that gene was related to the one found to be characteristic of the subgroup; the tumour was classified into a given subgroup when the cumulative score observed for the different signature genes was found to be the highest. The robustness of the subgroup was evaluated by the percentage of tumours that were correctly classified according to the defined signatures.

### The validation data set

To further validate these molecular subtypes, we used an external and independent tumourset, which included 97 tumours from the van't Veer et al. [10] and 12 tumours from the Sorlie et al.'s [4] microarray studies (Additional File 1, Table S5). These tumours were selected on the basis of the availability of expression data concerning the 47-gene set. In order to allow comparison between the Q-RT-PCR and the microarray data, the two data sets were median-centered independently. The thresholds for the analysis of the microarray data were defined as corresponding to those used for the Q-RT-PCR data analysis by using the QQ plots. We calculated quantile values for the Q-RT-PCR and microarray data (from the 1st percentile to the 100th percentile by step of 5%). Then, we set a function that linearly interpolated the quantile distributions. Using this function, given a Q-RT-PCR threshold, we could determine the corresponding microarray threshold. In the validation set, each tumour was assigned to one of the previously defined subgroups on the basis of the highest score it obtained through the different subgroups.

## Results

### Gene set selection

We selected 47 candidate genes from the published litterature and genomic databases. Most of these genes (see Additional File 1, Table S6, for the list of genes and their accession numbers) were chosen as likely to be involved in breast tumour sensitivity to steroid hormones. They included ERα target genes, which are either up- or down-regulated by oestrogen (Table 2), genes that specify the already reported breast cancer molecular subtypes (i.e. luminal, basal, normal-like and ERBB2), and genes that have been previously shown to be involved in sensitivity to the anti-oestrogen tamoxifen. As ERα activity has been shown to be regulated by cross-signalling with growth factor transduction pathways, we included also growth factor receptor and signalling genes. Moreover, the selected gene set also included some putative stem cell markers and genes coding for cell cycle regulators, because these genes are believed to contribute to tumor aggressiveness. We hypothesized that our selected set of genes would allow discriminating tumours according to both their hormone-susceptibility and aggressiveness. We hoped that by clustering tumours on the basis of the expression of these genes we could define new breast cancer subtypes.

### Hierarchical clustering of the gene expression profiles

**Figure 1:**
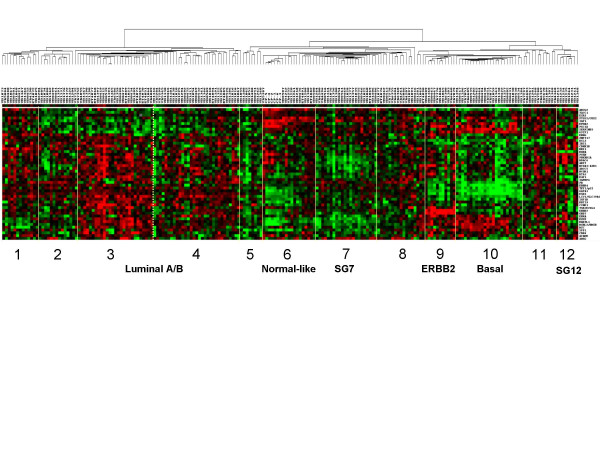
**Unsupervised analysis of the Q-RT-PCR expression data by pairwise hierarchical clustering**. 12 distinct subclasses were defined from the observed gene clusters. The luminal A/B, normal-like, ERBB2 and basal tumour subsets were identified according to gene expression signatures that have been previously reported to specify these molecular subtypes [2-4]. Subgroups 7 (SG7) and 12 (SG12) are also indicated.

Expression of the 47 genes was assessed by Q-RT-PCR amplification in the 199 breast tumours and 6 normal breast tissues. Normalized data were analysed in an unsupervised manner using a pairwise hierarchical clustering [13]. We used this classical approach to obtain a general description of how the selected genes co-varied with respect to their expression levels within the breast tumour population [14]. Thus, we determined 12 molecular subgroups that were characterized by a relative over-expression or under-expression of distinct combinations of genes (Figure 1). We limited the number of subclasses to avoid groups with too few samples that could hinder the reliability of any classification.

To assess the reliability of the clustering, we computed an average expression profile (i.e. a core subtype profile) for the tumours in each of the selected subgroups as performed by Sorlie and co-workers [3]. We calculated the Pearson's correlation of each sample to each of the 12 core subtype profiles. As illustrated on Figure S1 (Additional File 2), for more than 75% of the tumours, the correlation was the highest with the expression profile of the subgroup containing that sample, stressing the relevance of the defined subgroups. At least four subgroups (subgroups 6, 7, 9 and 10) appeared to be highly homogeneous since most of their tumors showed a correlation of 0.6 to 0.8 with their average subgroup profile.

Some of these subgroups were reminiscent of groups that have been previously reported [2-6]. For example, subgroup 10 gathered breast tumours in which the *GSTP1 *and *SERPINB5/maspin *as well as the *MAD2L1 *and *MYC *genes, which specify basal-type adenocarcinomas, were over-expressed (Figure 1). Moreover, in these tumours, genes, which have been shown to be over-expressed in luminal-type breast tumours [3,4] (see below), were under-expressed. Subgroup 9 comprised tumours that belonged very likely to the ERBB2-like subtype, as they overexpressed the *ERBB2 *and *GRB7 *genes. Interestingly, in subgroup 6, the 6 normal breast tissues (called CP) clustered together with a group of tumours that overexpressed *IGF1*, a feature which is characteristic of normal-like tumours. In contrast to previous reports, where other sets of genes were used [3,5,6], we were unable to clearly discriminate between luminal A and luminal B subtypes. Indeed, ER+ tumours were scattered in subgroups 1 to 4 that are characterised by the over-expression of a cluster of genes, which includes *CCND1*, *KRT19*, *IGF1R*, *LIV1*, *ESR1*, *GATA3*, *TFF1/pS2*, *ERBB4*, *PR *and *IGFBP4*.

On the other hand, our 47-genes set allowed us to define new molecular subclasses, such as the subgroups 7 and 12. Subgroup 12 was characterized by the up-regulation of the *PTEN*, *PRKAR1A*, *HDAC6 *and *AKT2 *genes, while subgroup 7 showed down-regulation of two groups of genes: the first one was constituted by the four genes cited above with the addition of *NCOA3*, *ABCC5*, *NCOR1 *and *E4F1*; the second included *GRB7*, *ERRA*, *EZH2*, *MAD2L1*, *MYBL2*, *MYC *and *SPP1*.

### Chi2 analysis of the identified breast cancer subgroups

To assess the statistical relevance of the molecular subgroups as defined by the hierarchical clustering, we performed a Chi2 analysis of the data (see Methods). This analysis allowed us to identify genes that were differentially expressed in one subgroup compared to the others and, therefore, to define a specific molecular signature for each subgroup.

As shown in Table 3, such specific molecular signatures could be assigned to 9 of the 12 previously defined subgroups. The genes of these specific signatures overlapped with the ones defined by the hierarchical clustering analysis. For example, among the 11 down-regulated genes of the signature of subgroup 10 (Table 3), 8 have been already observed in the cluster of down-regulated genes defined by the hierarchical clustering (namely *IGF1R*, *LIV1*, *ESR1*, *GATA3*, *TFF1/pS2*, *ERBB4*, *PR *and *IGFBP4*, see Figure 1). Also, the 6 genes, which specify subgroup 7, included 5 under-expressed genes (*ABCC5*, *AKT2*, *EZH2*, *HDAC6 *and *PRKAR1A*) that had been identified before by the hierarchical classification of the expression data (Figure 1).

The robustness of each subgroup was evaluated by the percentage of tumours in that subgroup that were correctly classified according to the defined molecular signature. As shown in Table 4, subgroups 2, 3, 7, 9 and 10 formed the most robust groups with over 80% of the tumours in each group showing the proper signature. Subgroups 1, 5 and 6 were found to be slightly weaker (with about 60–70% of tumours showing the specific signature). Subgroup 12 was found to be much less significant with only 43% of tumours classified correctly. Finally, a definitive molecular signature could not be assigned to subgroups 4, 8 and 11. However, a high proportion of tumours from group 4 (approximately 40%) exhibited the molecular signature that specified subgroup 3. Consequently, we decided to bring together subgroups 3 and 4 for the rest of the study.

### External validation of the molecular subgroups

**Figure 2:**
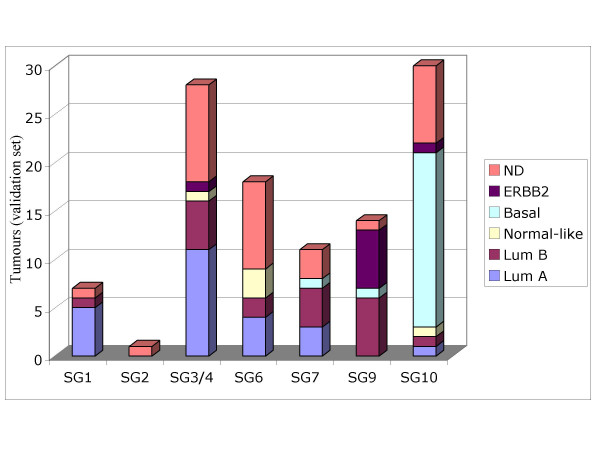
**Classification of tumours from an independent validation set according to the molecular signatures that specify the defined subgroups**. The validation set (109 tumours) included 24 luminal A, 19 luminal B, 5 normal-like, 8 ERBB2, 20 basal and 33 unclassified tumours. None of the independent tumours were classified into subgroups 5 and 12 as defined by hierarchical clustering and Chi2 analysis.

To further validate these molecular subtypes, we used an external and independent dataset that included 97 from the van't Veer [10] and 12 tumours from the Sorlie's [4] microarray studies (see Additional File 1, Table S5, for the list of these tumours). Each tumour in the validation set was assigned to one of the defined subgroups according to the highest score obtained by this tumour through the different subgroups. Accordingly, these external tumours were classified into 7 of the 9 subgroups that were defined following the Chi2 analysis (Figure 2). Among the 109 tumours used, 76 had been previously classified into the five reported molecular subtypes (i.e., luminal A, luminal B, basal-like, ERBB2, and normal-like), while 33 remained unclassified. As expected, the majority of the ERBB2 tumours (6 out of 8) were classified into subgroup 9, while the majority of the basal-type tumours (18 out of 20) were classified into subgroup 10. The luminal-type tumours were dispersed in different groups, confirming that our set of genes does not allow an optimal clustering of these tumours. The few normal-like tumours of the validation set were mainly assigned to subgroup 6. Finally, subgroup 7 apparently gathered together tumours that were previously classified into different molecular subtypes.

### Bioclinical features of the molecular subtypes

To adress the question of a possible clinical relevance for our classification, we first focused on the bioclinical features of the tumours from the 9 subgroups that were defined as robust by the Chi2 analysis. As shown in Table 5, subgroup 10 (basal subtype) included 90% of the ER- tumours with a high histological grade (86% SBRIII). As expected, the rate of recurrence in this group of tumours was among the highest (29%). Subgroup 10 (ERBB2 subtype) also included high SBR grade tumours (90% SBRIII), although these were both ER- (50%) and ER+ (50%). Similar observations were recorded, when the classification of external tumours was considered (Table 6). Indeed, subgroup 10 (which includes most of the basal-like tumours) and subgroup 9 (which includes most of the ERBB2 tumours) both exhibited a bad prognosis (with rates of recurrence of 57% and 53%, respectively) in agreement with their higher histological grade (80–100% SBRIII).

**Figure 3:**
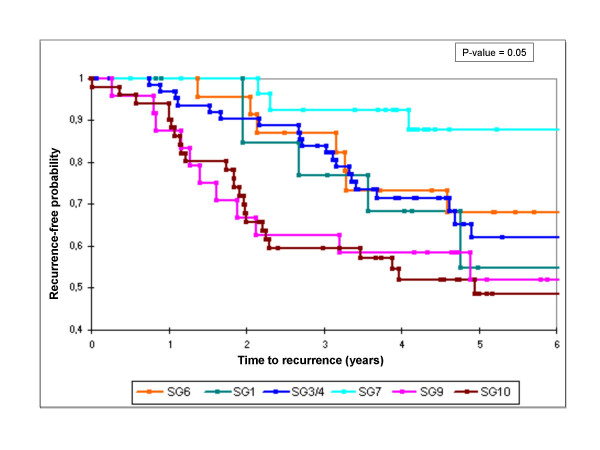
**Analysis of the recurrence-free probability in the subgroups defined according to Chi2 molecular signatures**. A Kaplan-Meier analysis was performed on tumours of the training and validation sets that were correctly classified in the indicated molecular subgroups. The *p *value was calculated by using the log-rank test.

Interestingly, the new tumour subclass (i.e. subgroup 7), which has been defined in this study, exhibited peculiar clinical features: tumours of this subgroup had mainly an ER+ status since it included 74% and 82% of the ER+ tumours of the training (Table 5) and validation (Table 6) sets, respectively; the percentage of pT1 tumours (< 20 mm) was higher in this subgroup than in the respective overall training (53% vs 29%, p = 0.06, Chi2 test) and validation (82% vs 52%, p = 0.04) cohorts. Finally, despite the fact that the patients were younger in subgroup 7 than in the overall training cohort (37% vs 18%, p = 0.06), we did not detect any recurrence within the 5 year follow-up (Table 5). Similar trends were observed in the validation setwith a lower recurrence rate in subgroup 7 than in the other subgroups (Table 6). To compare the time of recurrence between the different subgroups, we used the Kaplan-Meier analysis on the training and validation cohorts. As shown in Figure 3, this analysis emphasized the fact that tumours of subgroup 7 had one of the best prognoses.

## Discussion

The 500-gene set, which has been initially used to define the five to six breast cancer molecular subtypes [2-4], consisted of genes that had a significantly greater variation in expression between different tumours than between paired samples from the same tumour. The aim of the present study was to classify breast tumours on the basis of the expression of a limited set of genes that have been selected on the basis of their putative involvement in tumour sensitivity and/or aggressiveness. We anticipated that such a distinct set of genes could cluster tumours in a different way than that described in the studies by Perou [2] and Sorlie [3,4], allowing us to define new molecular subtypes. Our expectation was that such subclasses would help us define novel phenotypic subsets of breast cancer with a distinctive clinical outcome. Indeed, the current taxonomy of breast carcinomas seems insufficient to allow the classification of all breast tumours. However, a series of evidences suggests that a molecular classification of cancers may be a powerful and promising way to overcome our inability to accurately predict the clinical behaviour of breast cancers. Such an approach is expected to tackle the extreme complexity of the genetic alterations that are observed in breast cancers. The molecular signatures should, thus, represent a prognostic factor of greater efficiency than those currently used, such as the lymph node status, tumour size, hormone-receptor status or histological grade.

The molecular subtypes and gene-signatures reported so far have been mostly defined *via *microarrays studies [2-6,10-12]. Although such an approach allows the most efficient analysis to classify tumors, Q-RT-PCR has some advantages over microarrays since it provides accurate, reproducible and sensitive quantification of mRNAs. Moreover, the quantification of a limited number of genes avoids the discrepancy due to the restricted number of samples (tumours) in comparison to the too many variables (genes), which is a major drawback in the microarray studies [15]. Moreover, recent reports suggest the possibility to quantify gene expression using tissue sections from paraffin-embedded blocks as biological material, predicting the generalisation of the quantification of RNA expression in the clinical practice [16,17]. While extensive gene expression profiling using microarrays is unlikely to replace the standard immuno-histochemical assessment in the hospital practice, customized Q-RT-PCR platforms may represent a more affordable alternative as a clinically useful assay to identify molecular signatures. Moreover, it is important to note that a Q-RT-PCR study [18] has recently confirmed the 70-gene prognosis signature obtained by van't Veer and collaborators with cDNA microarrays [10]. Similarly, a real-time Q-RT-PCR assay has been recently shown to recapitulate the microarray classification of breast cancers [19]. Also, Q-RT-PCR has been used to quantify the expression of candidate genes in breast tumours of patients treated with tamoxifen[16] or chemotherapy [17].

The 47-gene set used in the present study was largely distinct from the 500-gene intrinsic subset selected by Perou et al. [2] and Sorlie et al. [3,4], and had only 15 genes that overlapped with that. Nevertheless, our minimal set of genes allowed us to discriminate the basal, ERBB2, normal-like and luminal subtypes, even though the luminal-type tumours were not tightly clustered but rather spread over several groups. Clearly, subgroups 9 (ERBB2 subtype) and 10 (basal subtype) were the more robust since most of the external tumours, which had been previously classified as ERBB2 and basal subtypes using the 500-gene intrinsic subset, were now assigned to subgroups 9 and 10, respectively. Indeed, 90% of the external basal-type tumours were classified into the subgroup 10 and 75% of the ERBB2 tumours were assigned to the subgroup 9. Subgroup 6 appeared to have a lower robustness as only 3 out of 5 of the external normal-like tumours were correctly classified in this subgroup. However, we would need a larger number of tumours from this subtype in the validation set to firmly conclude on the robustness of subgroup 6.

By contrast, our 47-gene set was clearly unable to discriminate between luminal A and luminal B tumours. As a consequence, tumours from the validation set, that have been previously identified as luminal A and B tumours, were not correctly classified in our study. This inadequacy could be due to the weak representation of genes from the 500-gene set in our own 47-gene set, since the use of sets of genes, which are different from the initial one, has been previously reported to be less efficient in discriminating the luminal A and luminal B subtypes [4,6]. Sorlie and collaborators [4] claimed that their inability to distinguish luminal A and luminal B tumours, when using the West's data set [20], was likely due to the fact that only half of the genes from their intrinsic gene list were found in this study. Furthermore, the luminal C subtype, which was initially reported by Sorlie in an earlier study [3], could not be reproduced [4] when using a separate 500-gene set (which had 200 genes in common with the former 500-gene set). The luminal A/luminal B distinction seems also less obvious in a recent study [6] that classified 83 breast tissue samples using a reduced set of genes, which included 120 genes from the later 500-gene set [4]. Last but not least, we failed to discriminate the luminal A/B tumours of the Sorlie's cohort on the basis of the 15 genes, which are shared by our 47 gene set and the 500-gene set, confirming that the size of the gene-set is likely to be a critical parameter.

However, our 47-gene set was able to define a new tumour group (i.e., subgroup 7). This new subclass, which we found to be relevant after internal and external validation, was shown to group together tumours with smaller size and a lower rate of recurrence, although a significant percentage of these tumours was ER negative and was from younger patients. This is true despite the fact that the training and validation cohorts were clearly distinct, as tumours studied by van't Veer et al. [10] (the majority of the tumours of our validation set) were from node-negative patients that were younger than 55 years and exhibited an overall high rate of recurrence. The fact that tumours of subgroup 7, from both training and validation sets, shared nevertheless some bioclinical features strengthens the accuracy of our classification with regard to this new subclass. Noteworthy, the molecular signature of subgroup 7 might represent a better prognostic factor than the histological grade, since it allowed low (training set) as well as high (validation set) SBR grade tumours to be classified with a better prognosis than the respective overall cohorts. On the other hand, as the tumours of subgroup 7 in the validation set were previously classified in different subtypes, one can hypothesize that these tumours were not well identified. Obviously, further studies using larger cohort of patients will be necessary to validate our findings.

In any case, breast cancer taxonomy needs to be improved and new tumour subclasses have to be defined. Molecular subtypes and signatures should be subsequently confirmed in prospective trials. Indeed, studies like ours do not consent to discriminate between prospective and predictive signatures since the majority of the patients receive adjuvant therapy, which, hopefully, will have an incidence on their clinical outcome. However, once clinically validated, tumours classifiers based on minimal molecular signatures should help therapeutic decision-making and treatment-tailoring for each patient.

## Conclusion

By studying the expression of 47 genes selected on the basis of their potential implication in breast cancer sensitivity, we have classified a cohort of 199 primary breast tumours into a series of molecular subgroups. The subgroup 7, which has been highlighted by our study, was remarkable as it grouped together mainly small ER+ tumours from rather young patients with a low recurrence rate. Although this finding should be confirmed on a larger cohort, it suggests that gene expression profiling using a minimal set of genes may allow the finding of new breast cancer subclasses with specific bioclinical features.

## List of abbreviations

Q-RT-PCR, quantitative reverse-transcriptase polymerase chain reaction.

## Competing interests

The author(s) declare that they have no competing interests.

## Authors' contributions

MC performed the RT-PCR study as well as the data and statistical analyses. HF and CR contributed to perform the biological study. CT and AH participated in the design of the biological and statistical studies, respectively. VN contributed to the statistical analysis. FB was in charge of the tumours' collection. JMD designed the study, supervised the data collection and data analysis and wrote the manuscript. All authors read and approved the manuscript.

## Pre-publication history

The pre-publication history for this paper can be accessed here:



## Supplementary Material

Additional File 1 **Supplementary Tables**, showing the post-operative treatments followed by the 199 patients of the studied cohort (**Table S1**), the histological types of the 199 tumours used in this study (**Table S2**), the bioclinical features of the tumours of the molecular subgroups as defined by hierarchical clustering of gene expression data (**Table S3**), the Chi2 values and thresholds corresponding to Chi2 > 15 (**Table S4**) and the bioclinical data concerning the tumours used for the validation set (**Table S5**). Click here for file

Additional File 2 **Supplementary Figure S1**, showing the correlation of individual tumour samples to the more representative core expression-based subtype profile. Click here for file

## Figures and Tables

**Table 1:** Chi2 value calculation

	Subgroup *k*	Other subgroups
Number of tumours with gene *j *expression ≥ threshold value	O_11_	O_12_
Number of tumours with gene *j *expression ≤ threshold value	O_21_	O_22_

Chi2 = N*(O_11_*O_22_-O_12_*O_21_)^2^/(O_11_+O_21_)*(O_12_+O_22_)*(O_11_+O_12_)*(O_21_+O_22_), with:

N = total number of tumours = O_11_+O_12_+O_21_+O_22_

O_11 _= number of tumours from class *k *whose gene *j *expression level was ≥ threshold value...

**Table 2:** Functional classes of the 47 selected genes

**Functional class**	**Genes**
Steroid hormone receptors and homologs	*ESR1, ESR2, PR, ERRA, ERRG, RXRA*
ERα target genes	
œstrogen up-regulated	*AREG*, *BCL2*, *CCND1*, *HDAC6*, *IGF1*, *IGFBP4*, *IRS1*, *KRT19*, *LIV1*, *MTA3*, *MYC ****PR***, *TFF1/pS2*, *TSK/E2IG4*
œstrogen down-regulated	*ABCC5*, *GSTP1*, *SERPINB5/maspin*
ERRα target genes	*ACADM*, ***TFF1/pS2***, *SPP1*
ERs/ERRs regulators	*NCOA3/AIB1*, *NCOR1*, *PGC1A*
Genes specifying molecular subtypes	
luminal A	***BCL2***, ***ESR1***, *GATA3*, ***KRT19***, ***LIV1***, ***TFF1/pS2***
luminal B/C	*MYBL2*
basal	***GSTP1***, *MAD2L1*, ***MYC***, ***SERPINB5/maspin***
ERBB2	*ERBB2*, *GRB7*
normal-like	***IGF1***, ***PGC1A***
Genes involved in tamoxifen responsiveness	*AKT2*, ***CCND1***, *CDKN1B*, *EPHA2*, *ESR2*, ***HDAC6***, ***IRS1***, ***NCOA3***, ***NCOR1***, ***PR***, *PRKAR1A*, *PTEN*
Growth factor receptor and signaling genes	***AKT2***, ***EPHA2***, ***ERBB2***, *ERBB4*, *IGF1R*, ***IRS1***, ***PTEN***
Cell cycle genes	***CCND1***, *CDK4*, ***CDKN1B***, *E4F1*, ***MAD2L1***
Stem cells markers	*ABCG2*, *BMI1*, *EZH2*
Others	*PTGS2/COX2*, *TACC1*, *ZNF217*

Genes are indicated in bold characters when present in an extra family.

**Table 3:** Molecular signatures specifying breast cancer subgroups as defined by hierarchical clustering and Chi2 analysis.

**SG1**	**SG2**	**SG3**	**SG5**	**SG6**	**SG7**	**SG9**	**SG10**	**SG12**
***ACADM***	*ERRA*	***BCL2***	*MTA3*	***ESR2***	*ABCC5*	*BCL2*	***ABCG2***	***AKT2***
***BMI1***	*IGF1*	***CCND1***	*SPP1*	***IGF1***	*AKT2*	***ERBB2***	*BMI1*	***CDKN1B***
***ERRG***	*NCOA3*	***CDK4***		*KRT19*	***AREG***	***GRB7***	*CDKN1B*	*E4F1*
*MYC*	*PTGS2*	***E4F1***		*MYBL2*	*EZH2*	*IRS1*	***EPHA2***	***EZH2***
***RXRA***		***ESR1***			*HDAC6*	***NCOA3***	*ERBB2*	***HDAC6***
		***GATA3***			*PRKAR1A*	***PGC1A***	*ERBB4*	*MAD2L1*
		*GSTP1*				***SPP1***	*ESR1*	***PR***
		***KRT19***					*GATA3*	***PRKAR1A***
		***LIV1***					***GSTP1***	***PTEN***
		***MAD2L1***					*IGF1R*	*RXRA*
		***MTA3***					*IGFBP4*	
		*PGC1A*					*LIV1*	
		*SERPINB5*					*PR*	
		*TSK/E2IG4*					***MYBL2***	
		***ZNF217***					***MYC***	
							***SERPINB5***	
							*TFF1/pS2*	

These signatures included up-regulated (bold characters) or down-regulated genes as indicated. No specific signature was found concerning subgroups 4, 8 and 11, except that a high proportion of tumours from group 4 exhibited the subgroup 3-signature (see Table 4).

**Table 4:** Percentage of tumours from subgroups 1 to 12 that show the best scores for the respective molecular signatures as defined by Chi2 analysis

**Subgroups defined by hierarchical clustering**												
	**1**	**2**	**3**	**4**	**5**	**6**	**7**	**8**	**9**	**10**	**11**	**12**
**1**	**62**	7	0	6	0	0	0	6	0	0	8	0
**2**	15	**86**	4	25	14	0	9	29	0	0	17	29
**3**	23	14	**96**	**41**	0	5	0	24	0	0	33	0
**5**	0	0	0	9	**71**	21	0	6	0	0	17	14
**6**	8	0	0	0	14	**58**	5	12	0	0	0	29
**7**	0	0	0	16	14	21	**86**	24	9	13	17	14
**9**	0	0	0	3	0	0	0	12	**82**	0	8	0
**10**	0	0	0	0	0	0	0	0	9	**88**	0	0
**12**	0	0	0	0	0	0	0	0	0	0	0	**43**

Columns represent the different tumour subgroups as defined by Eisen's hierarchical clustering. Rows are related to the distinct molecular signatures determined by Chi2. The percentage of tumours from Eisen's subgroups that exhibited proper molecular signatures are highlighted in bold. The sum of the % from each column may be higher than 100% as some tumours could exhibit extra signatures. As 41% of tumours from subgroup 4 exhibited the molecular signature that specified subgroup 3, tumours from subgroups 3 and 4 were assembled for the rest of the study.

**Table 5:** Bioclinical features of the tumours of the molecular subgroups as defined by hierarchical clustering and Chi2 analysis

Subgroup	Number of tumours	Hormonal status		Age	Tumour size		Lymph node status		Histological grade (SBR)			Clinical outcome
	n	% ER+	% ER-	% <50 years	% pT1	% pT2-3	% pN0	% pN1	% SBRI	% SBRII	% SBRIII	% recurrence

1	8	88	12	13	38	50	38	50	25	75	0	25
2	12	100	0	8	25	75	67	17	25	58	17	8
3/4	38	95	5	11	21	76	53	42	8	74	16	13
5	5	80	20	0	20	80	60	40	20	80	0	0
6	5	40	60	0	20	80	60	40	40	40	20	20
7	19	74	26	37	53	42	47	42	53	47	0	0
9	10	50	50	10	40	60	50	50	0	10	90	20
10	21	10	90	29	24	71	57	43	5	5	86	29
12	3	100	0	67	0	100	67	33	0	33	67	33
Overall cohort	121	70	30	18	29	68	54	40	18	49	31	15

Only tumours from the Eisen's subgroups, which were correctly classified according to the Chi2 defined molecular signatures, were considered in this study (i.e. 121 out of 199). Data related to subgroup 6 did not take into account the normal breast tissues. The sum of the percentages for a given subgroup may be less than 100% as tumor size, histological grade or lymph node status were occasionally not determined.

**Table 6:** Bioclinical features of the tumours of the validation set forming the molecular subgroups as defined by the Chi2 analysis

Subgroup	Number of tumours	Hormonal status		Age	Tumour size	Histological grade (SBR)			Clinical outcome
	n	% ER+	% ER-	% <50 years	% <20 mm	% SBRI	% SBRII	% SBRIII	% recurrence

SG1	7	100	0	71	43	0	71	29	43
SG2	1	100	0	100	100	0	0	100	0
SG3/4	28	93	7	39	46	18	39	43	46
SG6	18	89	11	67	78	22	39	39	28
SG7	11	82	18	73	82	27	9	64	27
SG9	14	57	43	64	36	0	0	100	57
SG10	30	30	70	70	40	3	17	80	53
Overall cohort	109	70	30	61	52	12	27	61	44
