# Supplementary material for: A new molecular breast cancer subclass defined from a large scale real-time quantitative RT-PCR study
Source: BMC Cancer. 2007 Mar 5;7:39. doi: 10.1186/1471-2407-7-39 (PMC1828062; doi:10.1186/1471-2407-7-39)
Supplement: Additional File 1 — Supplementary Tables, showing the post-operative treatments followed by the 199 patients of the studied cohort (Table S1), the histological types of the 199 tumours used in this study (Table S2), the bioclinical features of the tumours of the molecular subgroups as defined by hierarchical clustering of gene expression data (Table S3), the Chi2 values and thresholds corresponding to Chi2 > 15 (Table S4) and the bioclinical data concerning the tumours used for the validation set (Table S5). [file 1471-2407-7-39-S1.doc]

Additional File 1, Supplementary Table S1 - Post-operative treatments followed by the 199 patients of the studied cohort

Hormonotherapy (HT), chemotherapy (Chem) or both treatments (Chem+HT) are indicated in cyan, orange and yellow, respectively. Subgroups 1 to 12 were defined by hierarchical clustering of gene expression data. AI, aromatase inhibitor; Tam, tamoxifen.

Additional File 1, Supplementary Table S2 - Histological types of the 199 tumours used in this study

The 199 tumours included 139 ductal (70%), 35 lobular (17.5%), 10 mixed ductal/lobular (5%) and 15 other (7.5%) adenocarcinomas. Ductal, lobular and ductal/lobular carcinomas are indicated in cyan, yellow and green, respectively. DCIS, ductal carcinoma in situ; IDC, invasive ductal carcinoma; ILC, invasive lobular carcinoma; ICC, intraductal comedo carcinoma; mPC, micropapillary carcinoma.

Additional File 1, Supplementary Table S3 - Bioclinical features of the tumours of the molecular subgroups as defined by hierarchical clustering of gene expression data

Sum of % SBR for one given subgroup may be less than 100% as histological grade was occasionally not determined.

Additional File 1, Supplementary Table S4 - Chi2 values and thresholds corresponding to Chi2 > 15

Chi2 values > 15 are indicated in cyan, thresholds specifying the signature genes are highlighted in yellow

Supplementary Table S5 - Bioclinical data concerning the tumours used for the validation set (mainly from the van't Veer’s study)

Tumours were classified into subgroups 1, 2, 3, 6, 7, 9 and 10 according to the molecular signatures described in the present study. Sorlie’s classification into luminal A (LA), luminal B (LB), ERBB2, normal-like and basal subtypes is indicated when available (unclassified tumours are indicated by a question mark).

Additional File 1, Supplementary Table S6 - List of the 47 genes and their accession numbers
