# Supplementary material for: A new molecular breast cancer subclass defined from a large scale real-time quantitative RT-PCR study
Source: BMC Cancer. 2007 Mar 5;7:39. doi: 10.1186/1471-2407-7-39 (PMC1828062; doi:10.1186/1471-2407-7-39)
Supplement: Additional File 2 — Supplementary Figure S1, showing the correlation of individual tumour samples to the more representative core expression-based subtype profile. [file 1471-2407-7-39-S2.ppt]

## Slide 1
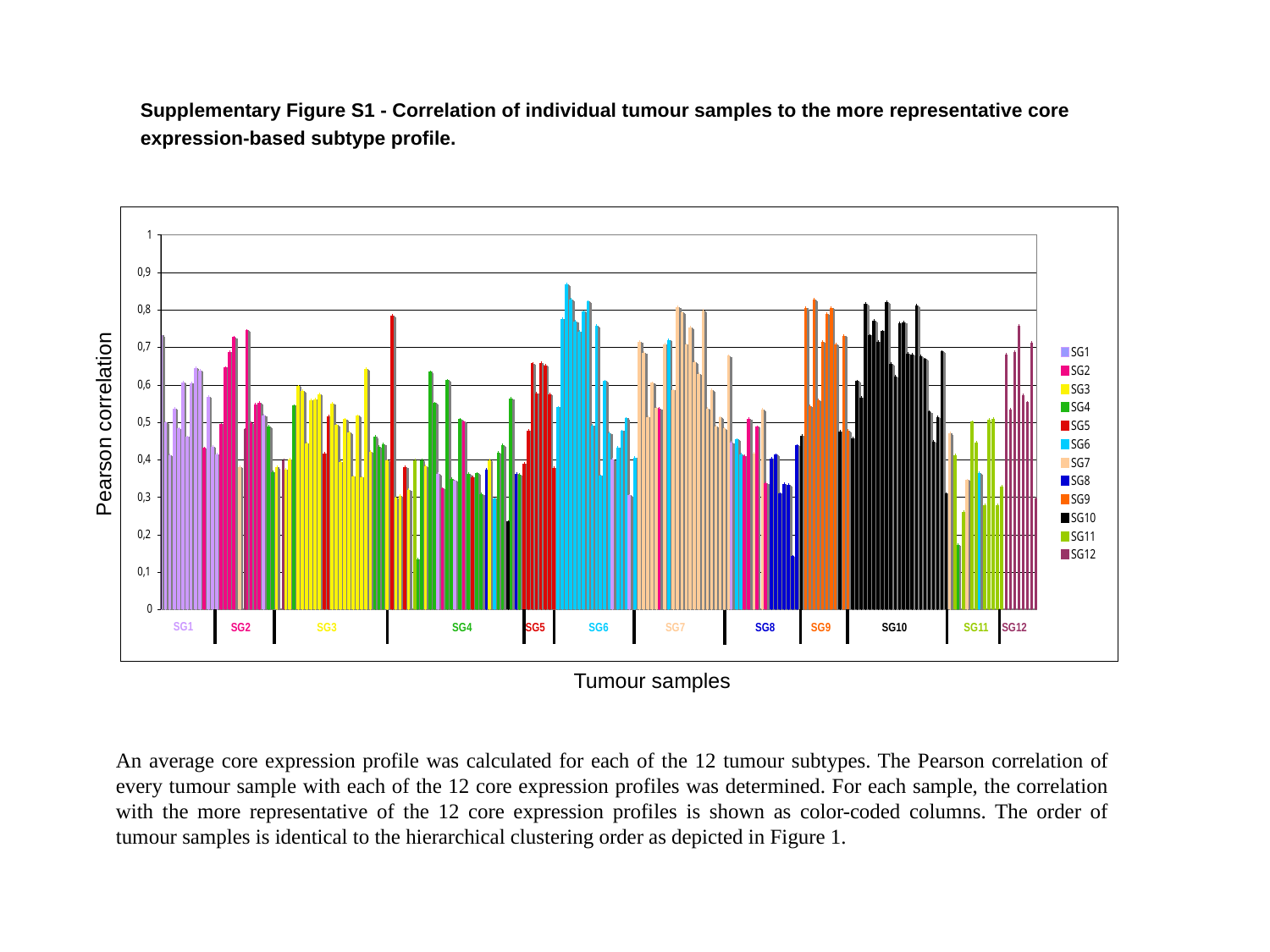

Supplementary Figure S1 - Correlation of individual tumour samples to the more representative core expression-based subtype profile.
Pearson correlation
Tumour samples
An average core expression profile was calculated for each of the 12 tumour subtypes. The Pearson correlation of every tumour sample with each of the 12 core expression profiles was determined. For each sample, the correlation with the more representative of the 12 core expression profiles is shown as color-coded columns. The order of tumour samples is identical to the hierarchical clustering order as depicted in Figure 1.
